# Altered ripple density inside seizure onset zone in patients with focal cortical dysplasia‐associated epilepsy

**DOI:** 10.1002/brb3.2169

**Published:** 2021-05-07

**Authors:** Cuiping Xu, Xiaohua Zhang, Guojun Zhang, Xiaoming Yan, Kai Ma, Liang Qiao, Xueyuan Wang, Xi Zhang, Tao Yu, Yuping Wang, Yongjie Li

**Affiliations:** ^1^ Beijing Institute of Functional Neurosurgery Xuanwu Hospital Capital Medical University Beijing China; ^2^ Department of neurology Xuanwu Hospital Capital Medical University Beijing China

**Keywords:** epilepsy surgery, focal cortical dysplasia, high‐frequency oscillations, stereoelectroencephalography

## Abstract

**Purpose:**

To evaluate the clinical and stereoelectroencephalography (SEEG) features and postsurgical outcome in a uniform series of patients who underwent epilepsy surgery and had pathologically confirmation of focal cortical dysplasia (FCD).

**Methods:**

We studied consecutive patients with drug‐refractory epilepsy who underwent SEEG recording. The high‐frequency oscillations (HFOs) features of SEEG, clinical characteristics, and surgical outcome were evaluated.

**Results:**

Sixty patients (31 FCD type I, 13 II, and 16 III) were analyzed retrospectively. Patients with type II tended to have their seizures at an earlier age than those with I and III (*p* < .01). Six different ictal onset patterns (IOPs) were identified. In patients with temporal lobe epilepsy (TLE), the most common patterns were rhythmic spikes or spike waves and LFRS, and in patients with extratemporal epilepsy, the most common patterns were low‐voltage fast activity (LVFA) and rhythmic spikes or spike waves. In addition, ripple density was found to increase significantly from the interictal to ictal onset sections and from the ictal onset to ictal evolution sections in patients with FCD I (*p* < .001). Regarding the distinct IOPs, ripple density continued to increase significantly between the interictal and ictal onset sections in LVFA, rhythmic spikes or spike waves, and burst of high‐amplitude polyspikes (*p* < .05). Ripple density decreased between ictal onset and ictal evolution sections in patterns of LVFA and rhythmic spikes or spike waves (*p* < .05). The mean follow‐up duration was 2.7 years (range 1–4.2), and 66.7% (*n* = 40) were class I. Patients with subtypes III and II had favorable surgical outcome than those with I.

**Conclusion:**

The clinical expression of seizure may depend on the pathological types with FCD II patients exhibiting their seizures at an earlier age. Distinct IOPs may demonstrate different ripple features and distinguishing the IOPs is very necessary to have an insight into the electrophysiological characteristics.

## Graphical abstract

Distinct IOPs demonstrate different ripples features. The clinical expression of seizure depend on the pathological types with FCD II patients exhibiting their seizures at an earlier age.

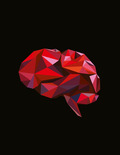

## 1 INTRODUCTION

Focal cortical dysplasia (FCD) is one of the most common pathological substrates of refractory focal epilepsy. It was first described by Taylor et al. (1971) The exact cause of FCD is unclear, but cellular and/or architectural abnormalities such as irregular neurons and balloon cells during utero brain development or early postnatal injuries are closely related to FCD (Krsek et al., 2010). According to the International League Against Epilepsy (ILAE) classification, FCD is classified into three pathological subtypes. The Magnetic resonance imaging (MRI) changes and electro‐clinical features of FCD subtypes are variable. High‐MRI resolution has improved the identification, and localization of some forms of FCD types, electrocorticography (ECoG), or stereoelectroencephalography (SEEG) provides invasive windows for understanding the intrinsic epileptogenicity of FCDs. More reports further suggested that high‐frequency oscillations (HFOs) occurring on SEEG were biomarkers of the epileptogenic zone (Kerber et al., 2013; Ferrari‐Marinho et al., 2015). In this study, we aimed to identify the electro‐clinical features in a cohort of patients due to FCDs.

## 2 MATERIALS AND METHODS

### 2.1 Patients selection

Between January 2016 and December 2017, 109 patients underwent SEEG and resective surgery in our epilepsy center. We retrospectively analyzed 63 patients with surgical resection of the epileptogenic region and pathologically confirmation of focal cortical dysplasia (FCD).

A comprehensive evaluation including detailed historical data, neurological examination, long‐term VEEG monitoring, and neuroimaging was performed among all the patients. In view of inconclusive or contradictive noninvasive evaluation, all the patients underwent SEEG with stereotactically placed intracranial electrodes to delineate the epileptogenic zone (EZ) precisely. The arrangement of multiple contacts electrodes (8–16 contacts with length 2 mm, diameter 0.8 mm, and 1.5mm apart) was designed individually according to the hypothesis of the EZ. The electrode position was confirmed by a postimplantation CT coregistered with the preimplantation MRI.

### 2.2 SEEG recordings and analysis

The SEEG signals were recorded on a 128‐channel Micromed EEG acquisition system (Micromed; Treviso, Italy) with a sampling rate of 512 or 1,024 Hz. During the monitoring of spontaneous seizures, the antiepileptic drug dosage was unchangeable. Intracerebral electrical stimulations were performed to map the eloquent cortex following the monitoring if necessary.

Five minutes of slow sleep segments during interictal periods were selected to detect high‐frequency oscillations (HFOs), which remained about 80‐250Hz. The automated detection of HFOs was performed according to the previous studies (Zijlmans et al., 2009). The ictal onset zone and ictal onset patterns (IOPs) were identified visually based on the careful observation of all SEEG traces. The ictal onset sections (4‐6s) and the seizure evolution (4‐6s) sections were selected from the ictal SEEG for HFOs analysis (Perucca et al., 2014). The IOPs‐specific changes in HFOs density were investigated across the three SEEG sections.

### 2.3 Postoperative follow‐up

The surgical effect evaluations were performed 6 months postoperatively and then annually. Surgical outcome was classified according to Engel's classification: (Engel et al., 1993).

(1) completely seizure‐free, auras only, or only atypical early postoperative seizures; (2) ≧90% seizure reduction or nocturnal seizures only; (3)≧75% seizure reduction; and (4) <75% seizure reduction. Seizure outcome at the last follow‐up visit was recorded for a general outcome overview.

### 2.4 Statistical analysis

Descriptive statistics were used for each variable among the three subtypes. The data were analyzed using one‐way ANOVA, independent sample *t* test and chi‐square test. Statistical analysis was performed using the SPSS 19.0 software package (IBM, Chicago, IL, USA). The significance level was set at 5% (*p* < .05).

### 2.5 Ethical statement

The study was approved by the Ethics Committee of Xuanwu Hospital, Capital Medical University, China, according to the Declaration of Helsinki. Written informed consent was obtained from all patients and their relatives.

## 3 RESULTS

### 3.1 Clinical data

Three patients were lost to follow up. Sixty patients (26 men and 34 women) were analyzed retrospectively in this study. The mean age at epilepsy onset was 10.3 ± 6.0 years (range 0.4–28), mean age at surgery was 23.9 ± 9.3 years (range 5–52), and mean epilepsy duration was 13.6 ± 9.0 years (range 1–46). The localization of the EZ was frontal in 20 patients, temporal in 23 patients, parietal in 4 patients, occipital in 3 patients, and 6 patients had more than one lobe involvement (one parietal‐occipital, 2 temporo‐occipital, 2 temporo‐parietal, and one fronto‐temporal), and 4 patients had insular involvement (one insular, 2 fronto‐insular, and one fronto‐temporo‐insular). Compared with FCD I and FCD III, FCD II patients tend to have their seizure at an earlier age (*p* = .008). The clinical data according to the FCD subtypes are summarized in Table 1, and Table 2 showed the detailed individual data.

**TABLE 1:** Clinical characteristics according to the FCD subtypes

Characteristics	Overall (*n* = 60)	FCD I (*n* = 31)	FCD II (*n* = 13)	FCD III (*n* = 16)
Male	26	13	8	5
Age at onset, yrs (range)	10.3 ± 6.0 (0.4–28)	11.2 ± 5.3 (2–21)	5.9 ± 5.1 (0.4–17)	12.1 ± 6.5 (2–28)
Age at surgery, yrs (range)	23.9 ± 9.3 (5–52)	24.5 ± 10.9 (5–52)	20.1 ± 5.7 (9–27)	25.8 ± 7.4 (13–40)
Mean epilepsy duration, yrs (range)	13.6 ± 9.0 (1–46)	13.3 ± 10.4 (1–46)	14.2 ± 7.3 (1–22.5)	13.6 ± 7.6 (1–28)
Follow‐up time, yrs (range)	2.7 ± 0.9 (1–4.2)	2.7 ± 0.8 (1–4)	2.8 ± 0.9 (1–4)	2.7 ± 0.9 (1–4.2)
MRI‐visible lesion (%)	25 (41.7%)	8 (25.8%)	7 (53.8%)	10 (62.5%)
Seizure outcome				
Favorable, Engel I(%)	40 (66.7%)	16 (51.6%)	10 (76.9%)	14 (87.5%)
Unfavorable, Engel II, III & IV (%)	20 (33.3%)	15 (48.4%)	3 (23.1%)	2 (12.5%)

**TABLE 2:** Individual patient profiles

Case No./Sex	Age at surgery (y)	Epilepsy duration (y)	Onset age (y)	Semiology	Interictal Scalp EEG	Ictal Scalp EEG	MRI lesion	SEEG IOPs	Surgery	FCD subtype	Engel class	Follow‐up (y)
1/F	9	5	4	L upper limb tonic/R limbs flexion and repetitive movement →pouting	R FCT	R FCT	P	Burst of high‐amplitude polyspikes	R F	FCD IIb	I	3
2/M	35	32	3	Shouting →head turning to R → L/R limbs clonic	L T	L FCPT	P	Theta/alpha sharp activity	L F	FCD Ib	III	4
3/M	22	5	17	R upper limb rising and tonic →L/R limbs tonic and clonic	B FT	L FT	P	Theta/alpha sharp activity	L F	FCD IIa	II	4
4/F	5	3	2	R limbs tonic →clonic of R facial →L/R limbs tonic and clonic	L FT	L FCT	N	Spike and wave activity followed by DEE (diffuse electrodecremental event)	L F	FCD I	I	3
5/M	11	2	9	Upper limbs rising (asymmetric tonic posturing)	R CPT	R CPT	P	LVFA	R F	FCD I	II	3
6/M	23	22.5	0.5	Trunk rocking back and forth	B F	B F	P	Theta/alpha sharp activity	R F	FCD IIb	I	3
7/F	26	20	6	R upper limb myoclonic	L FC	L FC	N	Rhythmic spikes or spike waves	L F	FCD IIb	I	3
8/M	25	10	15	L upper limb tonic →head turning to L → L/R limbs tonic and clonic	R FCT	‐	P	‐	R F	FCD I	I	2
9/F	13	1	12	Head turning to R and head clonic	L FCP	L FCP	N	Rhythmic spikes or spike waves	L F	FCD IIa	I	3
10/M	18	12	6	Ictal pouting →upper limbs rising (asymmetric tonic posturing)	R FCT	R F	P	LVFA	R F	FCD IIb	I	2
11/M	26	14	12	R limb repetitive movement/L upper limb tonic →lower limbs pedaling	B FCP	R FC	N	Rhythmic spikes or spike waves	R F	FCD IIa	I	3
12/F	12	2	10	Upper limbs rising (asymmetric tonic posturing) → cluster of spasm	L FP	L FP	N	Burst of polyspikes	L F	FCD Ia	I	2
13/F	13	5	8	L upper limb myoclonic	R H	R CPOT	N	Slow‐wave or baseline shift followed by LVFA	R F	FCD Ib	IV	2
14/F	7	1	6	Trunk rocking back and forth	L FT	L FT	P	‐	R F	FCD Ib	IV	2
15/M	25	23	2	Upper limbs rising →L hand clonic →humming	L T	L T	P	Theta/alpha sharp activity	R F	FCD IIb	II	2
16/F	20	10	10	R upper limb tonic →head turning to R → L/R limbs tonic and clonic	B H	B H	N	Burst of polyspikes	L F	FCD Ib	III	2
17/F	23	8	15	L upper limb tonic →head turning to L → L/R limbs tonic and clonic	B FCPT	R FCPT	N	Burst of polyspikes	R F	FCD Ib	III	2
18/F	27	14	13	R limbs tonic	B FCP	NEA	N	Slow‐wave or baseline shift followed by LVFA	L F	FCD Ib	I	2.8
19/F	20	19.6	0.4	R upper limb tonic →head turning to R → L/R limbs tonic and clonic	B FC	L FCP	P	LVFA	L F	FCD IIb	I	1
20/M	18	11	7	Upper limbs rising (asymmetric tonic posturing)	B H	NEA	N	Rhythmic spikes or spike waves	R F	FCD IIb	I	2.5
21/F	40	28	12	Shouting →head turning to R→ GTCS	L FCT	L FPT	P	Rhythmic spikes or spike waves	L P	FCD IIId	I	4.2
22/M	27	20	7	Dialeptic	R TP	R T	P	Slow‐wave or baseline shift followed by LVFA	R P	FCD IIa	IV	4.0
23/F	42	30	12	L upper limb tonic →head turning to L → shouting →GTCS	R T	R CPT	N	Theta/alpha sharp activity	R P	FCD Ia	I	3.0
24/F	18	14	4	L upper limb rising and tonic →R upper limb automatic behavior	NEA	NEA	P	LVFA	R P	FCD Ib	I	2.7
25/M	13	4	9	Dialeptic	R OT	R O	P	Rhythmic spikes or spike waves	R O	FCD IIId	I	4.0
26/F	16	1	15	Dialeptic →blinking	L T	L T	P	Rhythmic spikes or spike waves	L O	FCD IIId	I	3.5
27/M	35	20	15	Bilateral upper limbs rising and clonic	R TP	R TO	N	LVFA	R O	FCD I	IV	3.0
28/F	13	4	9	Head turning to L → L upper limb rising	R FT	R FT	N	LVFA	R TP	FCD I	IV	4.0
29/M	18	8	10	Head turning to L → L upper limb tonic and clonic →GTCS	B FT	B FT	N	Burst of polyspikes	R PO	FCD I	I	3.8
30/M	28	26	2	L upper limb rising and tonic →L upper limb clonic →head turning to L → GTCS	R TPO	R CPT	P	LVFA	R TP	FCD I	IV	4
31/F	19	8	11	dialeptic →Head turning to R → R upper limb tonic and clonic	B FT	L TO	N	Burst of polyspikes	L TO	FCD Ic	I	2.5
32/F	22	13	9	Dialeptic →blinking	L TPO	L TPO	N	LVFA	L TO	FCD Ic	IV	3.4
33/M	21	20	1	Chapeau de gendarme→R upper limb rising and tonic	B T	NEA	N	LVFA	L FI	FCD IIb	I	4
34/F	18	12	6	Upper limbs rising (asymmetric tonic posturing)	R F	NEA	N	LVFA	R I	FCD I	I	4
35/M	41	23	18	Oral automatisms	R T	NEA	P	Theta/alpha sharp activity	R FTI	FCD Ib	I	3.5
36/F	24	10	14	R upper limb tonic →humming	L F	NEA	N	Theta/alpha sharp activity	L FI	FCD Ib	I	3
37/M	25	10	15	Head turning to R → R upper limb rising and tonic →R limbs tonic and clonic →GTCS	L FCPT	L FC	N	LVFA	L FT	FCD Ib	II	3
38/F	19	12	7	R upper limb tonic→Head turning to R	L FTP	L FTP	N	Slow‐wave or baseline shift	L T	FCD IIIa	I	2.5
39/F	27	25	2	Dialeptic	B FT	R T	N	LFRS	R T	FCD IIIa	I	2.3
40/F	32	20	12	R hand covering mouth→L hand automatisms	L T	L FT	P	Slow‐wave or baseline shift followed by LVFA:3	L T	FCD IIIa	I	3.0
41/M	25	13	12	L upper limb tonic →L upper rising and tonic→Head turning to L→ GTCS	R T	NEA	N	LFRS	R T	FCD IIIa	I	2.0
42/F	24	12	12	Head turning to R → B upper limbs tonic →R upper limb clonic →GTCS	B T	L T	N	LVFA	L T	FCD IIIa	I	3.0
43/M	28	12	16	Retching →R upper limb tonic →head turning to R	L T	L T	P	Rhythmic spikes or spike waves	L T	FCD IIIa	II	2.9
44/M	24	6	18	R upper limbs tonic	L T	L T	P	LFRS	L T	FCD IIIa	IV	2.9
45/M	26	12	14	L upper limbs tonic →oral automatisms	L T	R T	N	Theta/alpha sharp activity	R T	FCD I	I	2.8
46/M	24	18	6	L upper limbs tonic and R upper limb automatic behavior →oral automatisms	L T	NEA	N	LFRS	R T	FCD IIIa	I	1.0
47/M	19	5	14	Oral automatisms	R T	R FT	N	‐	R T	FCD Ic	IV	2.4
48/F	40	22	18	L upper limbs tonic →humming → B upper limbs tonic →L limbs tonic and clonic	B T	R T	P	LVFA	R T	FCD Ib	I	2.3
49/F	52	46	6	R hand automatisms	B T	R T	N	Rhythmic spikes or spike waves	L T	FCD Ic	IV	2.3
50/F	37	17	20	L hand automatisms →B upper limbs tonic and clonic	L FT	L T	N	Theta/alpha sharp activity	L T	FCD Ia	IV	2.2
51/F	38	10	28	Oral automatisms and L hand automatisms	R T	R T	P	Theta/alpha sharp activity	R T	FCD IIIa	I	2.1
52/F	29	8	21	stare blankly →L hand automatisms	R T	R T	N	Rhythmic spikes or spike waves	R T	FCD Ib	I	1.0
53/M	32	26	6	L upper limbs rising and tonic	R H	R FCT	N	Rhythmic spikes or spike waves	R T	FCD I	I	1.0
54/F	33	20	13	Oral automatisms →R upper limb tonic→stare blankly	R T	R T	P	LFRS	L T	FCD IIIa	I	1.8
55/F	27	7	20	B hands automatisms	NEA	R PO	P	Rhythmic spikes or spike waves	R T	FCD IIIa	I	1.7
56/F	23	20	3	Stare blankly →B hands automatisms	B FT	R TPO	N	Theta/alpha sharp activity	R T	FCD IIIa	I	1.6
57/M	22	4	18	B hands automatisms	B H	L T	N	Burst of high‐amplitude polyspikes	L T	FCD Ib	II	1.5
58/F	13	11	2	R hand automatisms →humming → Trunk rocking back and forth	R FTP	R FT	N	Burst of high‐amplitude polyspikes	R T	FCD IIb	I	1.5
59/F	19	10	9	Shouting →R upper limbs L FT L FT rising and tonic →Head turning to R → GTCS			P	LVFA	L T	FCD IIIa	I	4
60/M	22	7	15	Oral automatisms	R T	NEA	N	Rhythmic spikes or spike waves	R T	FCD Ic	I	3

Abbreviations: B: bilateral; C: central; F: female; F: frontal; FCD: Focal cortical dysplasia; GTCS: generalized tonic‐clonic seizure; H: hemispheric; I: insular; IOP: ictal onset pattern; L: left; LFRS: low‐frequency rhythmic spikes; LVFA: low‐voltage fast activity; M: male; N: negative; NEA: no epileptic activity; O: occipital; P: parietal; P: positive; R: right; T: temporal.

### 3.2 SEEG ictal onset patterns

Two patients did not have any seizure during the SEEG monitoring, and one patient had seizures originating with diffused slow waves, which had limited value to delineate the epileptogenic zone (EZ). The three patients were excluded from further analysis. We finally identified six different IOPs. The most prevent pattern was rhythmic spikes or spike waves (25.0%), and then the second was low‐voltage fast activity (LVFA) (23.3%), then the third was theta/alpha sharp activity (18.3%), the forth was burst of polyspikes (15.0%), and then the last two were slow‐wave or baseline shift (10.0%) and LFRS (8.3%).

In patients with TLE, the most common patterns were rhythmic spikes or spike waves (27.3%) and LFRS (22.7%), and in patients with extratemporal epilepsy, the most common patterns were LVFA (28.9%) and rhythmic spikes or spike waves (23.7%). Four IOPs, rhythmic spikes or spike waves, theta/alpha sharp activity, slow‐wave or baseline shift, and LVFA were shared by each FCD subtypes.

LFRS were found only in FCD III, and burst of polyspikes was found in FCD I and II.

### 3.3 Features of high frequency oscillations

**FIGURE 1:**
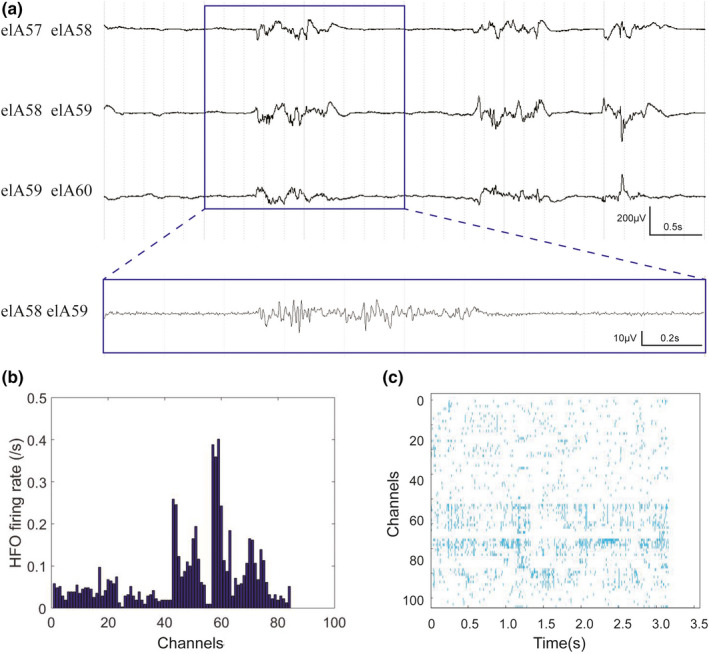
Representative interictal SEEG pattern of a patient with FCD I. (a) Unfiltered SEEG showing fast activity in the contacts. The blue part in A is expanded in time and amplitude, showing ripple in the figure below. (b) The histogram demonstrating the ripple rate. The contacts (elA 57–59) showed the higher rate. (c) The raster showing the ripple density

**FIGURE 2:**
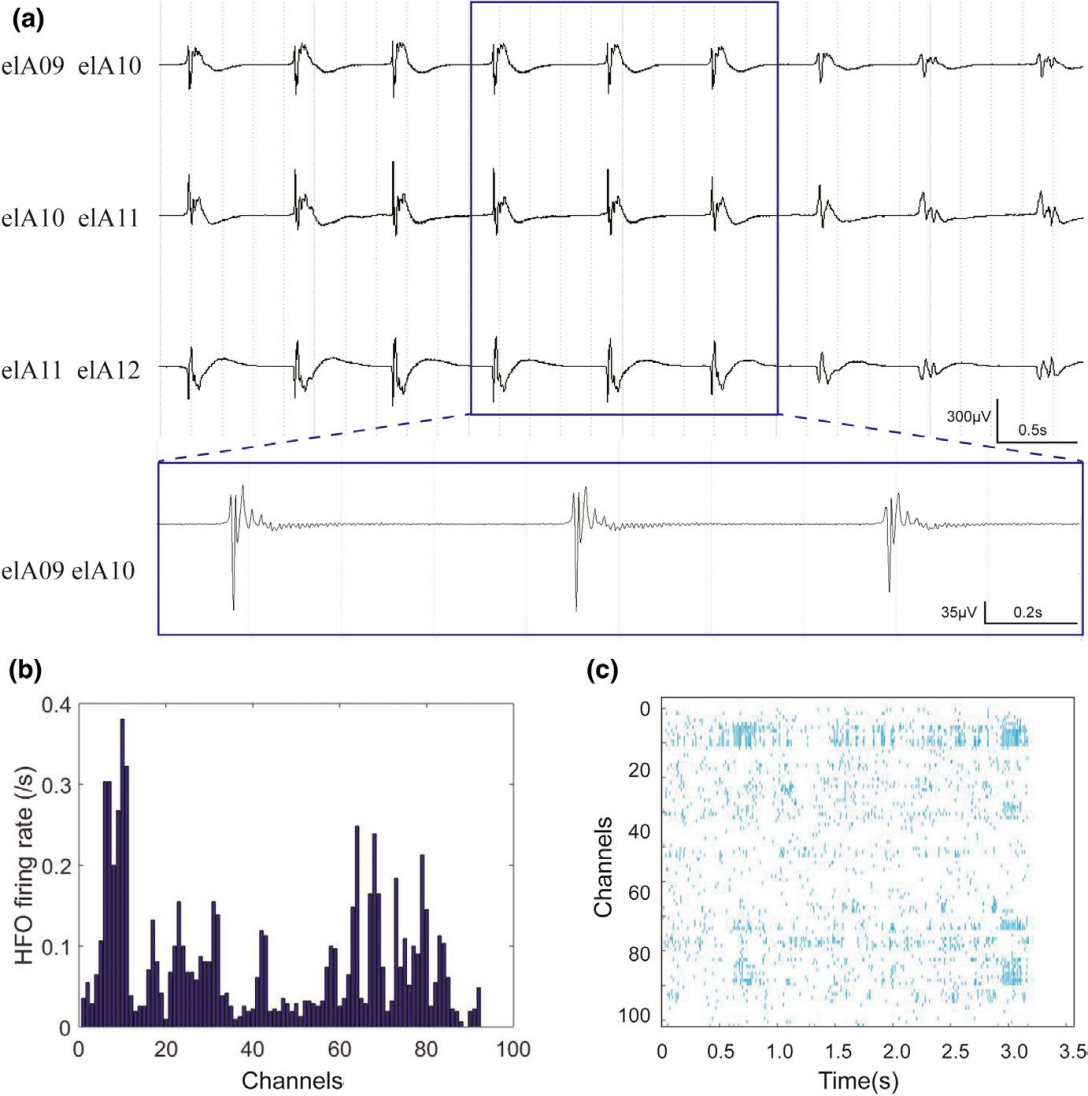
Representative interictal SEEG pattern of a patient with FCD II. (a) Unfiltered SEEG showing fast activity in the contacts. The blue part in A is expanded in time and amplitude, showing repetitive ripple in the figure below. (b) The histogram demonstrating the ripple rate. The contacts (elA 9–11) showed the higher rate. (c) The raster showing the ripple density

**FIGURE 3:**
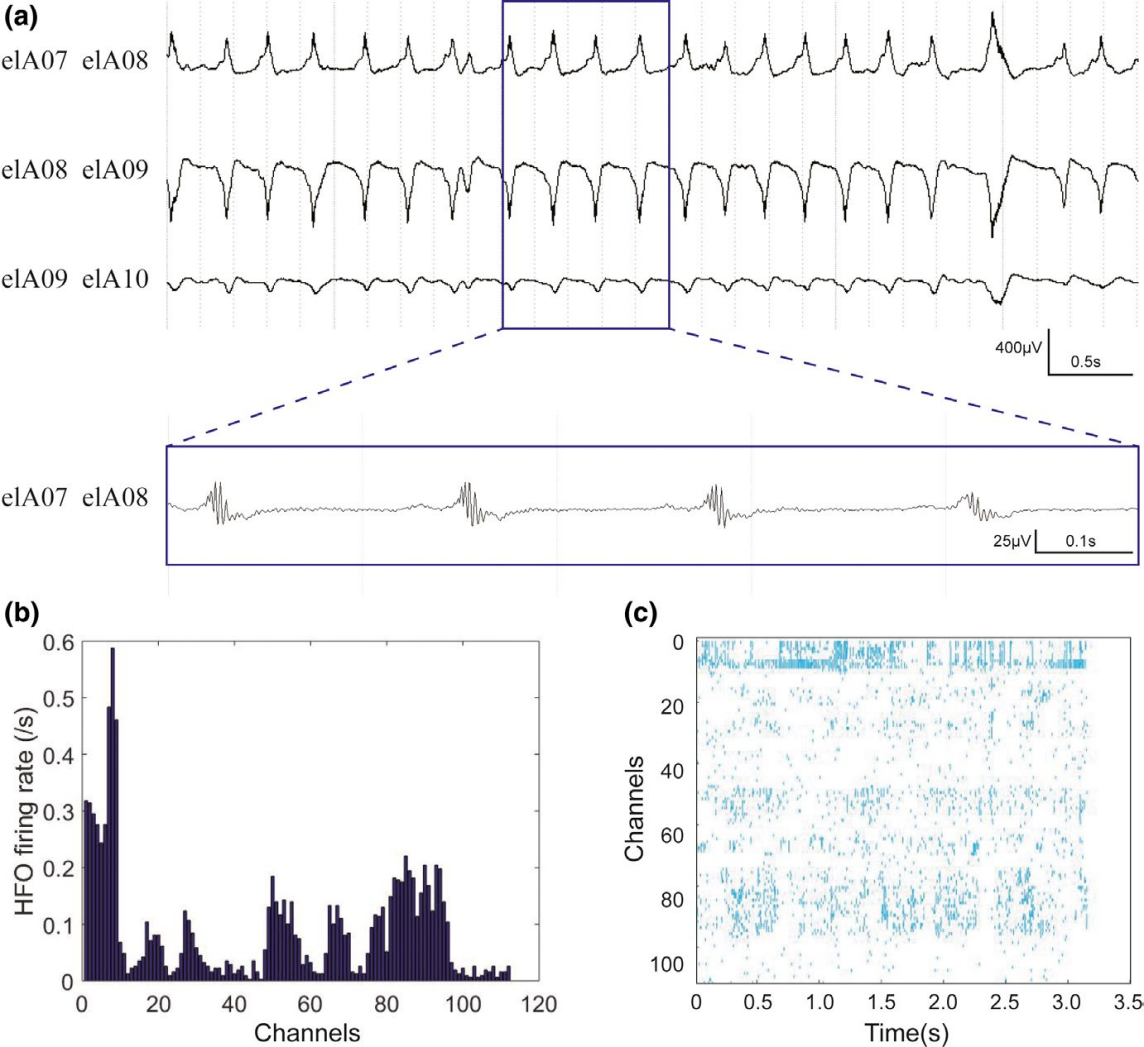
Representative interictal SEEG pattern of a patient with FCD III. (a) Unfiltered SEEG showing fast activity in the contacts. The blue part in a is expanded in time and amplitude, showing repetitive ripple in the figure below. (b) The histogram demonstrating the ripple rate. The contacts (elA 7–9) showed the higher rate. (c) The raster showing the ripple density

Forty‐three patients with sampling rate of 1,024 Hz were analyzed for ripples. Figures 1, 2, 3 showed the representative interictal SEEG patterns, ripple rate, and ripple density.

**FIGURE 4:**
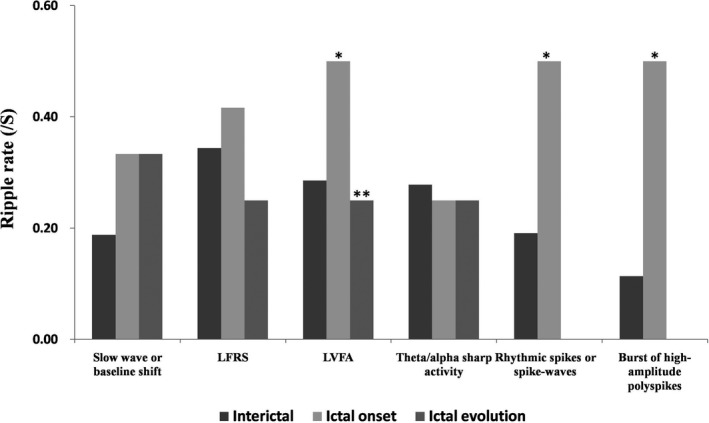
Ripple rate of each of IOPs. In the patterns of LVFA, rhythmic spikes or spike wave and burst of high‐amplitude polyspikes, ripples inside the SOZ increased significantly between the interictal and ictal onset sections. In the patterns of LVFA and rhythmic spikes or spike waves, ripples inside the SOZ dropped between the ictal onset and ictal evolution sections. * compared with the interictal sections; ** compared with the ictal onset sections

In patients with FCD I, ripple density inside SOZ was found to increase significantly from the interictal to ictal onset section, and decrease from the ictal onset to ictal evolution section (*p* < .001). These findings did not apply to both FCD II and III subtypes. Figure 4 demonstrated the ripple rate of each of IOPs. In LVFA, rhythmic spikes or spike waves and burst of high‐amplitude polyspikes, ripple density continued to increase significantly between the interictal and ictal onset sections (*p* < .05). In LVFA and rhythmic spikes or spike waves, ripple density decreased between the ictal onset and ictal evolution sections (*p* < .05). In slow‐wave or baseline shift and theta/alpha sharp activity, ripples remained relatively stable after ictal onset. In LFRS, ripple density was found to decrease between ictal onset and ictal evolution, but the change did not reach statistical significance.

### 3.4 Surgical, pathological outcomes and prognosis

The mean follow‐up duration was 2.7 years (range 1–4.2). The postoperative outcome was Engel class I for 40 patients (66.7%), Engel class II for 6 patients (10.0%), Engel class III for 3 patients (5.0%), and Engel class IV for 11 patients (18.3%). Pathological diagnoses consisted of FCD subtype I in 31 (50.8%) patients, type II in 13 (23.8%) patients, and type III in 16 (25.4%) patients. Sixteen patients (51.6%) with FCD I were Engel class I, 10 patients (76.9%) with FCD II were Engel class I, and 14 patients with FCD III (87.5%) were Engel class I. 25 (41.3%) patients had MRI‐visible lesions (eight histopathologically proven FCD type I; 7 type II; ten Type III). Ten patients (32.3%) with frontal EZ and 9 patients (29.0%) with temporal EZ were FCD I. In patients with FCD II, 76.9% (10 of 13) were frontal EZ and 81.3% (13 of 16) were temporal EZ in patients with FCD III.

## 4 DISCUSSION

This study characterized the clinical and SEEG features in a cohort patients with histopathologically proven FCDs. Isolated FCDs were found in frontal, temporal, parietal, occipital, and insular lobes, but multilobar FCDs were located preferentially in parietal‐occipital, temporo‐occipital, and temporo‐parietal. The frontal lobes were prone to present with FCD II, and the temporal lobes were prone to present with FCD I and II. Compared with subtypes I and III, type II patients have their first seizure at an earlier age. This confirmed that the occurrence and prevalence seem to be less dependent on the pathological types except the type 2b FCD (Najm et al., 2014). Less than 50% patients had MRI‐visible lesions, and the positive rate was lower in patients with FCD I than other subtypes. Surgical resection was the best treatment, and the surgical outcome depended on the histopathological subtypes. Patients with FCD subtype II and III had favorable outcome, and patients with FCD type I had the less favorable prognosis.

We found six distinct SEEG IOPs. Among these patterns, rhythmic spikes or spike waves and LVFA were the most common patterns. To assess the identified patterns across the different anatomical location of seizure onset, we classified the patients into temporal lobe group and extratemporal group. Patients with TLE were more presenting with rhythmic spikes or spike waves and that with FLE were more showing LVFA. Four IOPs, rhythmic spikes or spike waves, theta/alpha sharp activity, slow‐wave or baseline shift, and LVFA were shared by each FCD subtypes.LFRS were found only in FCD III, and burst of polyspikes was found in FCD I and II.

HFOs, which consist of ripples and fast ripples, have been considered as an indicator of seizure onset zone (Crepon et al., 2010; Jacobs et al., 2009). Ripples appeared in the broader areas than fast ripples. It is challenging to identify that which ripple generator areas were the target for resection and the changes in HFOs between interictal and ictal recordings. In this study, we only analyzed the ripples because of the sampling rate limitations during the monitoring. The findings were that ripples increase at ictal onset and decrease at ictal evolution in patterns of LVFA and rhythmic spikes or spike waves, and the significant increase was found at ictal onset in pattern of burst of high‐amplitude polyspikes. Rhythmic spikes or spike waves and burst of high‐amplitude polyspikes belongs to hypersynchronous (HYP) onset patterns. Both of LVFA and HYP are the main IOPs. Higher rates of ripples were associated with LVFA‐onset pattern seizures and HYP‐onset pattern seizure. A recent study by Salami et al. suggests that ripples reflect population IPSPs generated by principal cells entrained by synchronously active interneuron networks and fast ripples reflect the HYP firing of principal (glutamatergic) neurons in patients with mesial temporal sclerosis. However, our findings suggest that patients with the same pathology (FCD) but with different ictal onset patterns could share the same mechanisms (Salami et al., 2015). After ictal onset, ripples remained relatively stable in two patterns (slow‐wave or baseline shift and theta/alpha sharp activity). These findings confirmed the suggestion that distinct IOPs may demonstrate different HFOs features (Lévesque et al., 2012). The LVFA‐onset pattern and HYP‐onset pattern could be used to help localize the seizure onset zone. So, distinguishing the IOPs is necessary in the analysis of ripples during the interictal and ictal events.

During the various stages of brain development, such as neuroepithelial cell division, neuronal migration, and differentiation, FCD may occur due to the developmental abnormalities (Najm et al., 2014). In 1971, Taylor first described FCDs as a malformation of the cortex that consisted of dysmorphic neurons and giant neurons. More recent studies focused on the mechanisms of epileptogenicity in FCD, and three major mechanisms were proposed, including alterations in synaptic functions, glial cell dysfunction, and extrasynaptic dysregulation. Palmini et al. (1995) and Boonyapisit et al. (2003) reported the intrinsic in situ epileptogenicity of FCD using intraoperative and extraoperative intracranial recording with subdural electrodes or SEEG recordings. Moreover, through the study of HFOs, Brazdil et al. (2010) further confirmed the in situ epileptogenicity of FCD. However, there is some debate about the various FCD subtypes. In patients with FCD IIb, showing "tuber" or "tumor‐like" high‐FLAIR signal intensities, the area within the rich balloon cell density displayed the least epileptogenicity and the surrounding area within the less balloon cell density displayed the significant epileptogenicty (Boonyapisit et al., 2003).

## CONFLICT OF INTEREST

None.

### PEER REVIEW

The peer review history for this article is available at https://publons.com/publon/10.1002/brb3.2169.
